# Assessing the performance of maternity care in Europe: a critical exploration of tools and indicators

**DOI:** 10.1186/s12913-015-1151-2

**Published:** 2015-11-02

**Authors:** Ramón Escuriet, Joanna White, Katrien Beeckman, Lucy Frith, Fatima Leon-Larios, Christine Loytved, Ans Luyben, Marlene Sinclair, Edwin van Teijlingen, and EU COST Action IS0907. ‘Childbirth Cultures, Concerns, and Consequences’

**Affiliations:** Directorate-General for Health Planning and Research, Ministry of Health of the Government of Catalonia, Barcelona, Spain; Department of Experimental and Health Sciences, Universitat Pompeu Fabra, Barcelona, Spain; Centre for Research in Anthropology/Centro em Rede de Investigação em Antropologia (CRIA-IUL, Lisbon, Portugal; Department of Health and Social Sciences, University of the West of England, Bristol, UK; Nursing and Midwifery research unit, University hospital Brussels, Vrije universiteit Brussel, Brussel, Belgium; Department of Health Services Research, The University of Liverpool, Liverpool, UK; Departamento de Enfermería. Facultad de Enfermería, Fisioterapia y Podología, Universidad de Sevilla, Sevilla, Spain; Zurich University of Applied Sciences, School of Health Professions, Institute of Midwifery, Zurich, Switzerland; Women’s Clinic, Spital STS AG, Thun, Switzerland; Maternal Fetal and Infant Research Centre, University of Ulster, Coleraine, UK; Centre for Midwifery, Maternal & Perinatal Health Bournemouth University, Bournemouth, UK

**Keywords:** Measurement, Tools, Evaluation, Quality indicators, Health services, Normal birth, Physiological birth

## Abstract

**Background:**

This paper critically reviews published tools and indicators currently used to measure maternity care performance within Europe, focusing particularly on whether and how current approaches enable systematic appraisal of processes of minimal (or non-) intervention in support of physiological or “normal birth”. The work formed part of COST Actions IS0907: “Childbirth Cultures, Concerns, and Consequences: Creating a dynamic EU framework for optimal maternity care” (2011-2014) and IS1405: Building Intrapartum Research Through Health - an interdisciplinary whole system approach to understanding and contextualising physiological labour and birth (BIRTH) (2014-). The Actions included the sharing of country experiences with the aim of promoting salutogenic approaches to maternity care.

**Methods:**

A structured literature search was conducted of material published between 2005 and 2013, incorporating research databases, published documents in english in peer-reviewed international journals and indicator databases which measured aspects of health care at a national and pan-national level. Given its emergence from two COST Actions the work, inevitably, focused on Europe, but findings may be relevant to other countries and regions.

**Results:**

A total of 388 indicators were identified, as well as seven tools specifically designed for capturing aspects of maternity care. Intrapartum care was the most frequently measured feature, through the application of process and outcome indicators. Postnatal and neonatal care of mother and baby were the least appraised areas. An over-riding focus on the quantification of technical intervention and adverse or undesirable outcomes was identified. Vaginal birth (no instruments) was occasionally cited as an indicator; besides this measurement few of the 388 indicators were found to be assessing non-intervention or “good” or positive outcomes more generally.

**Conclusions:**

The tools and indicators identified largely enable measurement of technical interventions and undesirable health (or pathological medical) outcomes. A physiological birth generally necessitates few, or no, interventions, yet most of the indicators presently applied fail to capture (a) this phenomenon, and (b) the relationship between different forms and processes of care, mode of birth and good or positive outcomes. A need was identified for indicators which capture non-intervention, reflecting the reality that most births are low-risk, requiring few, if any, technical medical procedures.

**Electronic supplementary material:**

The online version of this article (doi:10.1186/s12913-015-1151-2) contains supplementary material, which is available to authorized users.

## Background

Validated, reliable indicators, and methodological tools for collecting such indicators, are essential for measuring maternal health care provision, performance and quality, enabling comparison at various levels and evaluating progress against defined targets. Moreover, the monitoring of indicators can lead to better understanding of how maternity health care services function, identify areas requiring improvement and can point to the need for necessary research [1–3]. The ways in which maternity and other forms of health care provision are measured using indicators and tools are inevitably conditioned by whoever is designing and conducting this appraisal, what the aims of this activity are, and what types of decisions will be taken as a consequence [4]. At the same time, indicators of any form often play a valuable role in prompting useful questions and stimulating informed debate [3].

The aim of this paper is to provide a critical review of existing published literature on the tools and indicators currently being used to measure the performance of maternity care within Europe, identify the dominant focus of existing measurements, and highlight any areas which are not being systematically examined. This paper is part of COST Action IS0907, “Childbirth Cultures, Concerns, and Consequences: Creating a dynamic EU framework for optimal maternity care” (2011-2014), which was established to share and analyse experiences across EU countries in order to advance scientific knowledge about ways of improving maternity care provision and outcomes for mothers, babies and families both within and across EU countries. The Action was founded on a ‘salutogenic’ approach/perspective, viewing health as a continuum and considering overall well-being as opposed to ‘absence of illness’ [4], and systematically pooled knowledge in areas such as complex analyses of the labour course, the reduction of unnecessary intervention in birth, the promotion of normal birth and other individualised outcome measures with the underlying aim of improving maternal and neonatal outcomes [5]. The work subsequently became part of the ongoing work of COST Action IS1405: Building Intrapartum Research Through Health - an interdisciplinary whole system approach to understanding and contextualising physiological labour and birth (BIRTH) (2014-).

The current review was based on the premises of the COST Action that it is essential to learn both within and across countries about “best practice” in maternity care, from a salutogenic perspective, while including the complexity of each system but also recognising that a physiological birth, which generally necessitates few, if any, medical interventions, is associated with good or positive outcomes. Given its emergence from COST Action IS0907 this paper inevitably focuses on Europe (the original COST EU focus was broadened slightly to facilitate the literature search). However, despite its restricted geographical reach, the review can be considered relevant to non-European countries (low, middle and high income) and regions as well due its focus It critically analyses what is being measured and hence what knowledge is being created, through current available tools and indicators, provides an overview of the focus of current maternity care measurement in Europe, identifies gaps and proposes some new measures for comprehensive maternity care and quality assessment, based on a salutogenic perspective focusing on optimal, positive processes and outcomes. By maternity care we refer to all formal care in relation to pregnancy, childbirth and the postpartum period as part of health service provision [6, 7].

Complete, high quality data are essential for the creation of evidence-based public policy in health care [8]. Comparison across European countries is a valuable exercise for informing and improving maternity care policy [9] and has already identified limitations in the current monitoring of ante-natal and post-natal care [10]. Further, recent systematic reviews of outcomes related to optimum and/or positive maternal and neonatal health and well-being have identified how less attention has been given to the measurement of factors that contribute to well-being and positive health outcomes [11, 12]. The development of a core outcome data set of “salutogenically-focused” outcomes for intrapartum research has been recommended [12]. In this current review we were particularly interested in analysing whether and how current tools and indicators enable systematic measurement of processes of minimal (or non-) intervention in support of physiological or ‘normal birth’ and associated outcomes. There is growing interest in the promotion of physiological processes around giving birth, given current rates of over-intervention and their outcomes [13], for example, argued for a system-level shift to a focus on the promotion of normal reproductive processes. This proposition has direct implications for the ways in which maternal care is measured.

### Indicators and tools to measure maternity care

This section describes the definitions of tools and indicators underpinning the present review.

#### Indicators

An indicator can be defined as a measure used to express the behaviour of a system or part of a system which is collected in a standardised manner so that comparable data can be used for analysis [14, 15]. Donabedian provided one of the earliest and most widely applied categorisations of health care indicators in relation to the assessment of care, emphasising the importance of examining structure, process and outcome [1, 16]. According to Donabedian, *structure indicators* are those which represent the necessary conditions for the delivery of a given quality of care. These include human, physical, financial and other resources available for service provision, such as total number of health providers or bed capacity per hospital. However these particular indicators do not ensure that appropriate processes are carried out or that satisfactory outcomes are achieved by the system, so cannot be directly related to subsequent care provision or the quality of care. *Process indicators* include the set of activities that take place within the service and how these are performed, such as rates of particular interventions and the use of protocols, thereby measuring the delivery of health care to the target population. Process indicators represent the closest approximation of how actual health care is provided and are the most clinically specific of the three types of indicators, but there is concern as to what degree these measures can be related to clinically desirable outcomes [15]; it has been argued that process measures are only as good as the evidence which links them to outcomes [16].

*Outcome indicators* Measure aspects attributable to the healthcare offered, and can be negative or positive, for example mortality rates, health status, or patient satisfaction. A particular challenge related to outcome indicators is that they may be influenced by factors other than the care provided; sufficient evidence is necessary to demonstrate that the quality of care independently contributed to the outcome. Satisfaction with services, for example, is a complicated outcome to measure given the various possible influences at play. It has also been argued that differences in outcome may be attributable to data collection methods and case mix as well as care provision, hence outcome indicators can be improved if efforts are made to standardize data collection and case mix adjustment systems are developed and validated [17]. The OECD proposed, for example, that indicator sets should be population-based and should strive to represent both the most important risk and client groups and the most essential interventions for these groups, be these preventive, curative or caring [15]. Measuring outcomes is a recognised means of monitoring and comparing care, often in order to identify any improvements required, while it is also acknowledged that outcome differences can be linked to the *process* of care [6]; the various categories of indicator are therefore inter-related.

The collective mix as well as type of indicators used to measure care is also important. All indicators are individually limited: any one value will only give a very specific, limited perspective of a wider situation, which may hide other significant factors related to the type/quality of care provided. Caesarean rate (CR) measurement, for instance, may indicate a reduction over time, which might be interpreted as a positive development, but considering this indicator in isolation may disguise the fact that instrumental births or other interventions increase concomitantly as the CR decreases. The use of a range of indicators has therefore been recommended [18] - a ‘balanced scorecard’ approach - which provides varied yet complementary insight into the overall system of care, with the various indicators employed measuring important yet diverse aspects [3].

#### Tools

A tool is an instrument for carrying out a particular function; in the context of this review an instrument for collecting information on maternity care performance. Tools contain a range of indicators; it has been argued that tool kits for measuring quality of care should specifically include process indicators, for example [16]. Much of the background literature on tools for measuring health care refers to ‘quality indicators’ (also known as performance indicators or quality measures). These may be structural, process or outcome indicators which track significant changes, in other words deterioration and/or improvement, within a specific area of care [19]. The monitoring of quality indicators, in other words, a continuous or systematic periodical measurement of these values, is understood to substantially enhance understanding of what is working well or not, where efforts for improvement should be targeted, and the evolution of any introduced changes either within a particular unit of analysis (e.g. hospital, region, country) or across units [20, 21].

## Methods

For the purpose of this review we used Donabedian’s definition of indicators [1]. We understood a tool to be a collection of indicators used as an instrument for collecting information about a particular aspect of the performance of maternity care, and if it was defined as a tool by the authors of the publication in question. This does not necessarily mean that it has been validated in practice (some of the papers reviewed propose a new tool or the validation of a tool).

### Criteria for selection of studies

As explained, this work is an outcome of the COST Action IS0907, hence its scope is limited to European countries. To that end, we considered studies, reports or databases containing tools or indicators for the measure of maternity care in Europe to be eligible for inclusion.

### Search strategy

A team of eight individuals was involved in this work, all of whom collaborated on COST Action IS0907 (see Acknowledgements). As a first step we defined a strategy to search for tools and indicators measuring maternal health care at national, regional and local level within European countries (Additional file 1). The search was limited to research databases (Centre for Reviews and Dissemination, OvidSP, Scopus), published documents in peer-reviewed international journals and indicator databases which included the measurement of aspects of health care at a national and pan-national level (OECD, Eurostat and Euro-Peristat). We restricted our search to sources published between 2005 and when the review took place, September 2013. For practical reasons the search was restricted to the English language (also following the assumption that much of the information relevant Europe-wide would have been published in English). The search was conducted using different combinations of a set of keywords: maternity, maternity care, obstetric health services, satisfaction, reproductive health services, reproductive care, evaluation, measurement, assessment, accessibility, equity, organisation culture, sustainability, cost-effectiveness, outcomes, results, outputs, deliverables, indicators. The PRISMA statement was employed for reporting results

### Quality assessment of included studies

The quality of included studies was assessed using an “ad hoc” critical evaluation tool (Additional file 2) based on the Critical Appraisal Skills Programme Checklist [22] (CASP). This tool was structured to assess the quality of the studies and systematic reviews identified. Each was appraised separately for relevance and quality by two members of the team, aided by a pre-tested scoring grid. The documents were scrutinised in terms of whether they provided a clear tool or indicator/s which had been applied to the measurement of maternity care within Europe, their proven (or validated) transferability to other settings (given the known specificities in the structuring of maternity care in different countries), and appraised and graded in terms of their overall quality.

### Details of ethical approval

This study was exempt from Ethics Committee review as it used publically available data.

## Results

**Fig. 1:**
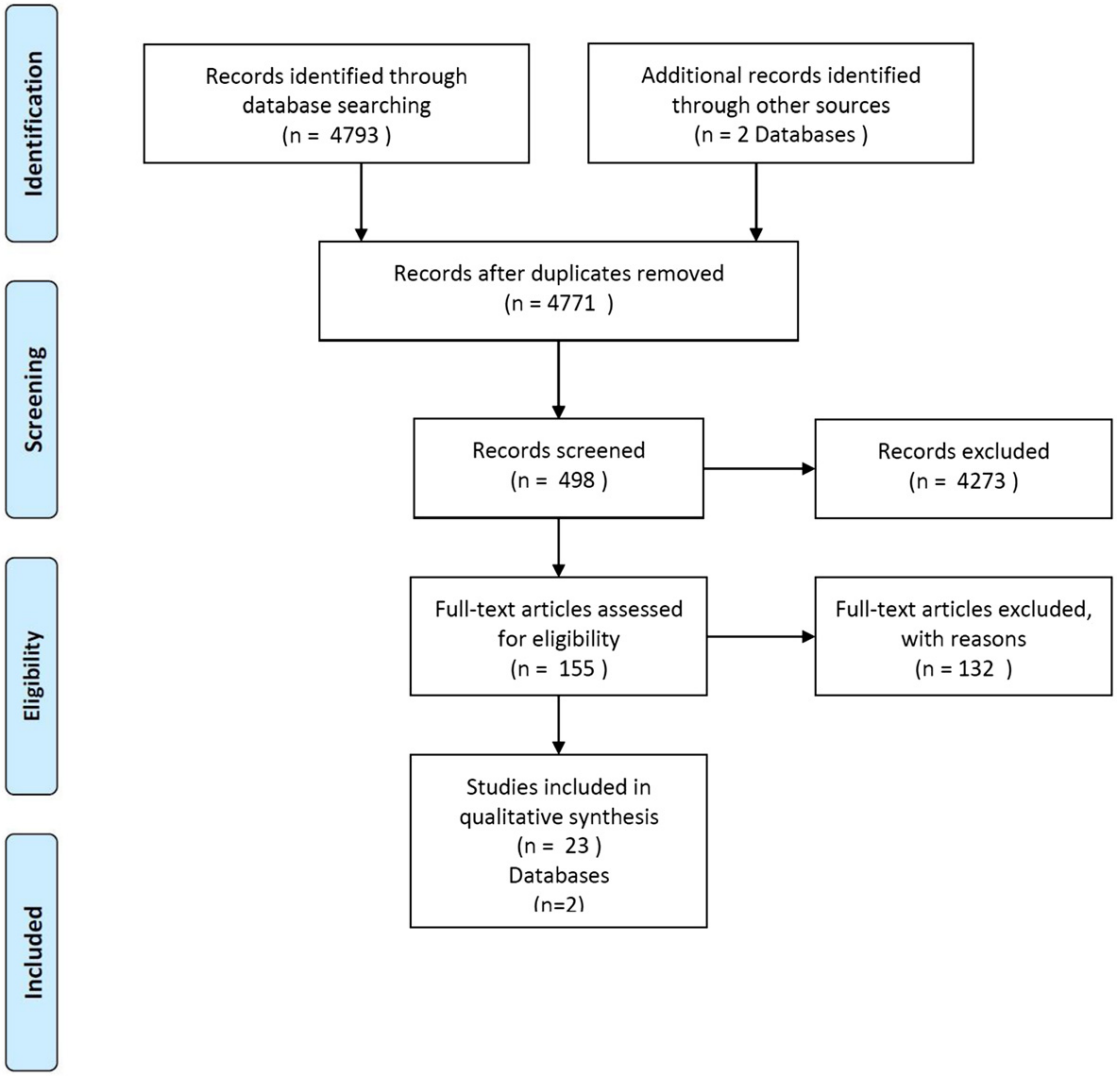
Flow-chart according to PRISMA statement

The final total of publications generated from the first-round selection was 498 references, plus two indicator databases (given that data are distributed by Euro-Peristat in summary form, we considered the latest Euro-Peristat report [23] as a published document. After close review of document titles and abstracts this selection was reduced to 155 sources. Of these, 110 were excluded due to unavailability (researchers could not locate a copy or the full document was only available in a language other than English), non-relevance to maternity care, or the source only related to a country/countries outside Europe. Out of the resulting 45 documents reviewed in detail, a further 22 documents were excluded, four because they related to non-European countries, thirteen because they did not include a tool or indicator/s, and three because they did not constitute a full study and two more because of poor methodological quality. This resulted in a total of two databases and 23 published references for detailed analysis [11, 19, 23-44]. Figure 1 is shown according to PRISMA statement for reporting results [45].

Table 1 shows a structured summary of the 23 published references and the two databases selected in terms of whether they constituted a tool or a set of indicators, and what particular area of care they were measuring. The selected references are also included in the References list at the end of the paper [[11, 19, 23-44]

**Table 1:** Summary of references selected

	Tool	Set Of Indicators	Structure			Process				Outcome				
			Material resources	Human resources	Models of maternity and organisation	Antenatal care	Intrapartum care	Postnatal care	Neonatal care	Antenatal care	Intrapartum care	Postnatal care	Neonatal care	Women’s satisfaction
Aniuliene R. et al, [24]		√			√									
Boulkedid R. et ol, [25]	√					√	√				√	√	√	
Bruin-Kooistra M [26]		√			√									
Chappel LCetaf, [27]		√					√				√	√		
Devane D. et ol, [7]		√		√	√	√	√			√	√	√	√	√
Euro-Pehstat Project with SCPE and Euro cat, [23]		√				√	√	√	√	√	√	√	√	
Eurostat. Database [28]		√		√	√						√			
Faisel H. et at, [29]	√										√		√	
Hollins-Martin C. et al, [30]	√													√
Knight H E. etal, [31]		√					√							
Murray SF. et al, [32]	√				√									
Nuti S. etal, [33]	√			√	√		√				√	√		√
OECD. Database [34]		√		√	√						√		√	
Overgaord C at ol, [35]	√													√
Parkhurst JO. et al, [36]		√	√	√	√	√					√	√		
Roosmalen J. et al, [37]		√				√	√					√		√
Rudman A et al, [38]	√										√			√
Sawyer A. et al, [39]	√(9 tools)													√
Sheridan M. et al, [11]	√				√	√					√	√	√	
Sibanda T. et al, [19]		√					√					√		
Tucker J. et al, [40]		√			√						√		√	
Turner M. J. [41]		√									√			
Voerman G. £ et al, [42]	√				√	√	√	√	√		√	√	√	
WHO Europe. Making pregnancy safer, [43]	√		√	√	√	√	√	√	√	√	√	√	√	
Wiegers TA, [44]	√			√	√									√

**Fig. 2:**
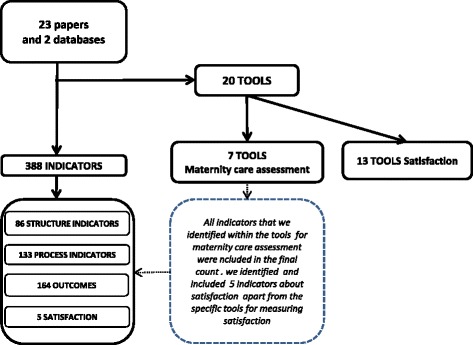
Flow-Chart of indicators and tools

The review uncovered a total of 388 indicators, 383 measuring structure, process and outcome and a further five measuring user satisfaction. Some of these indicators were grouped within tools (a total of seven tools). In addition, 13 distinct tools were identified which exclusively measured satisfaction with maternity care (Fig. 2). Although indicators of user satisfaction are generally considered within the category of care outcomes we chose to analyse tools related to satisfaction and their associated indicators separately in a different paper (now forming part of the work of COST Action IS1405) due to the methodological and analytical complexity of this topic, discussed in more detail below.

Both the Organisation for Economic Co-operation and Development (OECD) and Eurostat databases include maternity care-related indicators. In 2005 both Eurostat and the OECD adopted the International Classification for Hospital Morbidity Tabulation (ISHMT) as a shortlist for statistical comparison of hospital activity by diagnostic categories and information about outcomes such as maternal and infant mortality as well as structural indicators concerning health care professionals (e.g. number of midwives and obstetricians) are provided by both databases. OECD provides accessible datasets at country level, including structural indicators on health care resources, health status outcomes (e.g. neonatal mortality, maternal mortality and perinatal mortality), process indicators on health care utilisation (e.g. hospital discharges and average length of hospital stay by diagnostic categories), and outcome quality indicators for childbirth (e.g. obstetric trauma, vaginal birth with/without instruments). Eurostat, similarly, provides data on hospital discharges by diagnostic category, health care professionals and maternal and infant mortality which can be extracted at both country and regional level.

The 383 indicators identified from all the selected sources, including the two databases, focused largely on process and outcomes in maternity care (a total of 297 indicators); structure indicators were least represented (Table 2). Intrapartum care was found to be the aspect of maternity care assessed most often through both process and outcome indicators, while the postnatal and neonatal care of mother and baby were the areas least appraised. A large number of process indicators relating to antenatal care were also identified. Within the two international indicator databases (OECD and Eurostat), the systematic collection of process and outcome indicators reflected elements of the standard administrative procedures for monitoring care in the various countries included.

**Table 2:** Typology of indicators identified

Type of indicators	Number of indicators
Structure	
Human resources	9
Models of maternity care and organisation	77
Process	
Antenatal care	45
Intrapartum care	67
Postnatal care	5
Neonatal care	16
Outcomes	
Antenatal care	12
Intrapartum care	99
Postnatal care	16
Neonatal	37
Satisfaction	
Tools	13
Indicators	5

Caesarean Section rate (CR) was found to be the most commonly measured event, followed by the type of instrument employed (vaginal birth), and postnatal maternal complications (Table 3). Maternal morbidity was found to be the most frequently applied indicator regarding fatal/undesirable outcome (not shown in table).

**Table 3:** The 10 most frequently measured events

Classification	Measured events	Type
1	Caesarean section	Outcome
2	Vaginal delivery with instrument (type of instrument)	Outcome
3	Maternal complications postnatal	Outcome
4	Perineal tears	Outcome
5	Method of infant feeding	Process
6	Induction augmentation labour	Process
7	Vaginal delivery without instruments (may include normal birth)	Outcome
8	Apgar	Outcome
9	Other NN complicatons	Outcome
10	Mode onset labour	Process

Some variability was identified in indicators ostensibly measuring the same intervention, highlighting the difficulties of cross-site comparison in maternity care. For example in the measurement of perineal damage, sources varied in their definition of ranges related to the severity of damage; in the measurement of labour induction, the definition of this intervention was also found to vary widely.

### General tools

The 20 tools identified fell into two categories, seven general tools which used quality indicators to monitor and compare maternity care provision, largely drawing on data from hospital level, and thirteen formulated solely to appraise user satisfaction with services. The aims and foci of the general tools identified are summarised in Table 4. Very few of the tools were found to be measuring normal birth or positive birth outcomes.

**Table 4:** Areas of user satisfaction presented in identified tools

Satisfaction aspects
Satisfaction with care
Antenatal care
Before labour
Labour and birth care
Couples’ perceptions of care during labour and birth
Different types of birth
Caesarean section under regional anaesthesia
Safety of practice and care
Care procedures
Perceived reality of care and subjective importance of each item
Postnatal care
Women's perceptions of interpersonal care
Information and decision making
Provided education
Satisfaction with service provision
Physical birth environment
Antenatal care by Midwife, General Practitioner and Gynaecologist
Care during ultrasound
Hospital stay
Postpartum care by Midwife, General Practitioner and Maternity Care Assistant
Neonatal screening
Overall pathaway of care

The Delphi method, an iterative process using several rounds of data collection and analysis to generate group consensus [39, 46] emerged as a common approach for developing tools for maternity care measurement; three of the seven general tools were generated using a version of the Delphi approach. The multi-disciplinary nature of the stakeholder groups involved in developing tools through this method varied, however. In all cases obstetricians as well as other technicians such as paediatricians and anaesthetists participated in the tool development. Midwives were only included in the development of two of the tools, while service users were included in one [47].

### Tools measuring satisfaction

Of the 13 tools found to be measuring user satisfaction one was a comparative review of existing satisfaction measures, which identified nine questionnaires and concluded that despite continuing interest in this area, few validated indicators exist for measuring satisfaction with care during labour and birth [39, 48]. While not all content of the tools for measuring satisfaction could readily be classified as indicators, the measures identified can be usefully classified into two areas: satisfaction with care and satisfaction with service provision (Table 4).

## Discussion

The review aimed to critically examine existing published literature on the tools and indicators being used to measure the performance of maternity care within Europe. Findings emerged regarding a range of issues, including the focus of current forms of appraisal, whereby normal birth, non-intervention more generally and positive outcomes are rarely measured, the lack of consensus regarding optimal care and its measurement, and methodological and systemic issues related to indicator development and registration.

### Focus on the clinical aspects of intrapartum care

A dominant focus on the measurement of intrapartum care was identified. Most indicators were clinically focused, while the postnatal care of the mother was found to be the area least measured. This finding underlines existing concern about the limited monitoring of the quality of postpartum care [49-51]. It must also be recognised that despite the comprehensive measurement of technical interventions identified, many of these may have been unnecessary [16].

Table 3 reveals the predominance of CR as an overall indicator; this indicator was present in all of the tools devised to monitor and compare maternity care, and its comprehensive application highlights the perceived value of measuring CR in comparing performance both within and across hospitals. Some discussion emerged in the literature reviewed regarding the application of this indicator, however. One source highlighted the importance of measuring the CR rates of particular institutions and comparing these with the ‘normal’ range of elective and emergency caesareans; hospitals found to be above the normal range might need to review pre-labour obstetric practices, for example. Approaches were identified in some sources whereby CRs were disaggregated in relation to a core indicator of total CR, such as rates of caesarean section before/after onset of labour [52] and proportions of elective and emergency caesarean section [53] providing more meaningful information about the performance of the system, including decisions associated with the use of caesarean section and their outcomes. Since the initial review was conducted a new approach has emerged from the World Health Organisation (WHO) to measure CR at hospital level, based on Robson classification [54].

### Importance of case mix analysis and limited disaggregated analysis of low-risk women

The importance of nuanced understanding of the particular contexts in which interventions take place, and in particular the various sub-groups of users to whom care is offered, was similarly highlighted. It was argued, for example, that knowledge of the case mix in different settings is essential to the full understanding of any indicators [3]. Only two of the seven tools identified attempted to directly address this problem, however. In one tool a homogenous group of ‘standard primaparae’ (a typology of low-risk women) was defined in order for indicators in relation to these users to be compared across sites; in another the examination of CR rates both before and during labour included a disaggregated sub-sample of low-risk women to enhance understanding of this measurement [53].

The non-inclusion of the specific, care pathway (process and outcome indicators) of low-risk women in most of the general tools identified is, indeed, striking. As noted above, it has been proposed that disaggregated indicator sets should be used to represent the most important risk and client groups and the most essential interventions (be these preventive, curative or caring) for these groups [15]. Low-risk women can be deemed both a key risk and client group for monitoring care, not least due to their numerical predominance on a national level, in addition to other high risk user groups which may inevitably require a greater level of technical intervention, including caesarean section. Tracking the care of low-risk women requires particular tailored indicators throughout pregnancy and intrapartum and post-partum, including an indicator for normal, physiological birth (non-intervention); such measurement was found to be lacking.

### Limited measurement of non-intervention and optimal outcomes and systemic implications

Birth requiring no instrumental intervention was only found to be readily measurable through the application of one of the seven general tools identified. Only through the combined analysis of two indicators presented in this tool (‘delivery was vaginal, not caesarean section’ and ‘delivery occurred without instruments’) would it be possible to define the occurrence of non-instrumental vaginal births [55]. In terms of overall indicators, however, vaginal birth (no instruments) was common (see Table 3).

Moreover, it emerged that apart from this measurement, few of the total 388 indicators identified were found to be appraising optimal or positive outcomes more generally (excluding satisfaction, which is examined separately, below). Six of the seven tools were found to capture certain positive outcomes, but with a particular focus on the condition of the perineum (degree of tears/whether intact) and breastfeeding (early initiation/ continuation/support).

It has been argued that one of the criteria for selecting quality indicators in terms of importance and relevance is whether they clarify consensus on the objectives of a system/organisation. The overwhelming focus on technical, clinical interventions amongst the indicators and tools identified suggests that either the objectives of the system/organisation are to provide technical intervention, or current quality indicators are failing to clarify consensus regarding the overall philosophy and objectives of care; key elements of the system are not yet being captured comprehensively.

A focus on adverse outcomes in maternal care appraisal, and the lack of appropriate measurements for monitoring non-intervention during pregnancy and birth in low-risk cases and in the absence of complications has been highlighted in previous reviews [6, 7, 12]. Similarly, the current review identified an emphasis on technical aspects of care rather than a consideration of the systematic measurement of different and varying care processes which may contribute to the mode of birth. This is despite the fact that it has been recognised for some time that the complex inter-relationship between separate elements of care are crucial to outcome [56, 57]. Moreover, the links between care processes and outcomes related to physiological birth and any mix of indicators related to optimal outcomes was also found to be lacking. Findings of the current review therefore confirm the need identified by previous authors [11, 12] for the measurement of factors that contribute to well-being and positive health outcomes, but not only in the area of intrapartum care but across the spectrum of maternity care, including postpartum processes.

*Feasibility* and the need to focus on the most measurable data as opposed to what might be the most effective/interesting information was an issue which emerged from the literature [41, 58-60], raising the question as to whether normal birth is not being measured due to the difficulties involved in generating consensus and effective tools and indicators for monitoring this mode of birth. At the same time, Chappell et al. [59] made a persuasive case for the development of ‘aspirational’ indicators related to normal or unassisted birth through wider consultation and representation, challenging the argument that certain indicators are problematic to collect. Indeed, it has been argued that the indicators which are made available are closely tied to the objectives and philosophy of the organisation in question, not merely attributable to the availability of data.

### Factors influencing the focus of maternity care measurement

Both the professional context and the national or local maternal healthcare models from which care measurement strategies emerge inevitably condition the focus of maternity care appraisal. The construction of indicators is known to depend upon who designs and manages the measurement process, their understanding of the philosophy and objectives of the care system in question, what the aims of this exercise are, and what decisions will be taken as a consequence of the findings [18]. While maternity care is usually provided by a range of professionals from different disciplines whose philosophy of maternity care may range from pathology to normality, and whose definition/s of quality service provision and approaches to measuring this may differ accordingly, current measures are predominantly clinical.

### Lack of consensus regarding optimal indicator trajectories and targets

The possible impact of differing perceptions regarding overall philosophy of care and associated assessment of services is reflected in the variability of the tools and indicators reviewed, which exposes the challenge of developing a systematic, transferrable approach of appraisal across different sites. For example, it is difficult to make meaning or practical use of indicators if they are ‘stand-alone’, without a clearly identified target value or indicator direction identified, yet this is a subjective issue. In one of the selected references where a tool for monitoring quality of care was presented, the stated unwanted direction of rate change for epidural analgesia use amongst women who delivered vaginally was ‘decrease’ [41] viewing the rise of interventions as favourable is a debatable objective. This problem is exacerbated by the fact that, as noted in several of the sources scrutinised, there is no consensus about what constitutes good, or optimal care, and therefore no agreed criteria against which progress should be measured in relation to these categories [43].

In some of the literature reviewed it was described how targets and thresholds are a complicated aspect of performance monitoring, requiring national and international development and should ideally reflect universally accepted standards [19]. The issue of consensus is central here. At a local level, the Delphi approach is understood by some authors as a popular, successful method for defining a set of indicators (tools) as it enables individuals in various locations and with different areas of expertise to be included anonymously, often without a physical meeting, which prevents the views of a minority from dominating the group [25]. However, as already noted, the current review found the involvement of non-clinicians in the delineation of tools through the Delphi method to be rare, and it is recommended that the development of data sets includes the involvement of clinically-based health care professionals, maternity health care researchers as well as users of maternal health services [4].

### Debatable value of rare events as an indicator

Maternal morbidity and maternal and perinatal mortality rates were validated indicators frequently included in the tools reviewed. The limitations of using such rare events as indicators in developed country settings have been observed, and it has been argued that these measurements lack sensitivity for assessing obstetrical care and, particularly in the case of mortality, such events can be uncontrollable and uncertain, regardless of health intervention [11]. Alternatively, it has also been argued that rare and significant events (adverse outcomes) can provide an important starting point for in-depth studies aimed at understanding key issues relating to the care system [3]. The focus of current measurements of maternity care on rare, adverse events yet the neglect of “normality” and optimal outcomes is, nonetheless, a marked contrast.

### Measuring satisfaction as an indicator

Satisfaction is a complex element of maternity care to measure. Studies have shown that in some cases women were satisfied with care even if this does not meet their previous expectations, for example [61]. Two discrete areas of satisfaction emerged from the literature included in this review: one relating to women’s perceptions of the care they received and the other associated with the structure of services, such as the care pathway during the course of pregnancy (Table 4). However, the full findings of the review process related to satisfaction will be analysed in a separate paper, as certain publications emerged from the original literature searches which did not explicitly fit the requirements of the present review (and hence were not included for detailed analysis) but nonetheless provided important, alternative methods for assessing the impact and outcomes of care provision as expressed by women. One tool not included in this current review, for instance, explored women’s perceptions of outcomes and quality of life over an extended period following birth [62], an approach which has important implications for care provision.

### Transferability of tools

We considered a tool to be transferable when the tool containing indicators was well defined and could be replicable in other settings, in other words the same item could be measured in the same way in another (indeed any) setting. Using transferable tools or indicators may help for comparing different settings or organisations and could contribute to the identification of areas requiring improvement. Many of the indicators and tools identified can readily be used at different levels of service provision, to measure the activity of individuals or teams of clinicians, at maternity unit or birth centre level, or at the level of hospital site. They could equally be applied and aggregated, including at national or inter-country level, to provide comparison. While non-European countries were excluded from the review as our aim was to examine tools being used to measure maternity care in European countries, much may be learned from adapting and piloting tools across different contexts. For example, our review elicited a tool, the Optimality Index, which was elaborated in the Netherlands [63], and adapted elsewhere, including the US, and subsequently transferred from there to the UK, for example. This tool provides an innovative method of focusing on the positive side of each indicator, instead of the negative side, combining results into an overall index. It should be noted, however, that all of the tools identified depend on standard administrative procedures regarding registration data and are only as effective as the administration system in operation. In some settings ‘deviant’ registration tendencies have been identified whereby certain interventions that are routinely performed are not registered (e.g. artificial amniotomy, the use of certain medication to stimulate the normal process of labour) or no data are registered in a situation when no technical procedure/intervention has been performed [64].

## Limitations of the study

The study was limited to countries within Europe. We are aware that there may be a wealth of unpublished literature on the topic examined, there may also be tools and indicators with great potential which have not yet been applied, and that, further, within certain European countries there may exist databases with a range of indicators and tools for measuring and comparing maternity service performance and quality within the national health system at different levels (e.g. Healthcare Commission UK, 2007) which may be transferable to other settings. However, for practical reasons, beyond examining the international databases already mentioned which are relevant Europe-wide, we narrowed our focus to internationally published, peer-reviewed literature available in English.

## Conclusions

The ways in which maternity and other forms of health care provision are measured are inevitably conditioned by whoever is designing and conducting this appraisal, what the aims of this activity are, and what types of decisions will be taken as a consequence [18]. Both the scope and focus of measurement can be linked to the overall philosophy of care of those planning and managing health services, what these stakeholders seek to find and monitor, and, indeed, what they are concerned with changing. The review identified an emphasis on technical aspects of maternity, particularly intrapartum care in Europe, rather than a consideration of the systematic or comprehensive measurement of care processes contributing to non-intervention and physiological (normal) birth. It was also found that the links between care processes and outcomes related to a normal mode of birth are not being measured.

It has been argued that one of the criteria for selecting quality indicators is whether they clarify agreement on the objectives of a system/organisation. The current focus of tools and indicators suggests that either the objectives of maternity care are predominantly technical (as opposed to the avoidance of unnecessary technical intervention), or current quality indicators are failing to capture key elements of maternal and newborn care comprehensively [13]. Normal birth is just one area identified which is not being systematically measured. Given that less attention has been given to the measurement of factors that contribute to well-being and positive health outcomes we identified the need for new quality indicators aimed at measuring non-intervention, optimal outcomes, and the possible relationships between different areas, based on the reality that the majority of births are low-risk and should require little technical intervention. This development would counter the existing focus and contribute to a ‘balanced scorecard’ approach [3] to providing varied yet complementary insight into the overall system of care, with indicators measuring important yet diverse aspects.

As well as the comprehensive measurement of physiological birth with no technical intervention, disaggregated by various risk groups, possible topic areas for new indicators could include the communication and overall support women and their families receive during and after birth, women’s satisfaction with services, post-natal well-being of both mother and child, and the strengthening of women’s capability to mother their babies. Aspects of post-partum care would be integrated within the indicator set, in recognition of the importance of a health continuum for mother and child. The collection of such data might be methodologically challenging for systems conventionally more focused on quantitative measurement, but would significantly enlarge understanding of the performance of maternity care.

The recommended approach could not only improve current measurement and management of maternity care, but also contribute to the broader consideration of maternity as a normal life event and not a condition which inherently requires medical intervention. As there is no apparent cross-stakeholder consensus about what constitutes good, or optimal maternity care (as evidenced by current indicators and tools for measurement), agreeing criteria against which progress should be measured in relation to the vearious categories may be problematic. Any attempt to transform existing measures and incorporate new indicators in order to bridge identified gaps would require wide-ranging consultation and representation to achieve consensus. It would be advisable for any such initiative to involve representatives of a range of stakeholder groups, including midwives and service users.

Finally, differing ideas about the philosophy of maternity care highlights the concomitant challenges of effecting change. Any new indicators defined would need to be included in standard registration processes, a procedure which is likely to pose its own institutional challenges and would need to be championed by policy-makers.

## Additional files

Additional file 1:
**Search string.** (PDF 168 kb) Additional file 2:
**Critical evaluation tool.** (PDF 378 kb)
