# Supplementary material for: Assessing the performance of maternity care in Europe: a critical exploration of tools and indicators
Source: BMC Health Serv Res. 2015 Nov 2;15:491. doi: 10.1186/s12913-015-1151-2 (PMC4631101; doi:10.1186/s12913-015-1151-2)
Supplement: Additional file 1: — Search string. (PDF 168 kb) [file 12913_2015_1151_MOESM1_ESM.pdf]

Search strings

Scopus

Mater* OR obstetric* OR reproductive "health servic*”" AND evaluation OR assessment OR quality indicators

OvidSP

Maternal Health Services [Mesh] OR maternal-child Health Centers [Mesh] OR Maternity services OR (matern* AND service* OR system*) and evaluation OR assessment OR quality OR sustainability OR accessibility AND evaluation indicators

Center for Reviews and Dissemination

Maternal Health Services [Mesh]
