# Supplementary material for: Assessing the performance of maternity care in Europe: a critical exploration of tools and indicators
Source: BMC Health Serv Res. 2015 Nov 2;15:491. doi: 10.1186/s12913-015-1151-2 (PMC4631101; doi:10.1186/s12913-015-1151-2)
Supplement: Additional file 2: — Critical evaluation tool. (PDF 378 kb) [file 12913_2015_1151_MOESM2_ESM.pdf]

Critical evaluation of tools and indicators to measure maternity care in Europe

PART I

Questions to be answered on the critical evaluation

1	Reference of the paper (name)	
2	Availability	
3	Type of document	
4	describes the paper a tool and/or indicator?	
	Comments	
5	International relevanc. Is tool or indicator applicable in other settings or countries	
	Comments	
6	Evaluator/s name or identification	
	Comments	
7	Language	
8	Has the tool been translated in other languages	
	Comments	
9	Level (on what level the tool/indicator is aplicable)	
	Comments	
10	Evaluation tool/indicator purpose/objective	
	Comments	
11	Which aspects of maternity care the tool measures?	
	Comments	
12	Does the tool measure aspects about health professionals?	
	Comments.	
13	Tool /indicators *(name of tool or list of indicators)*	
14	Type of information to be collected in the tool	
15	Information source to construct tool or indicator	
	Comments	
16	Is the evaluated document of good quality	

Critical evaluation of tools and indicators to measure maternity care in Europe

PART II

QUALITY OF THE PAPER: APPRAISING THE PAPERS			TOTAL POINTS: STUDIES	0
Checklist for STUDIES (NO = 0; YES = 1) some questions include the opcion : don't know or not applicable				
complete this checlist when evaluating STUDIES	*FOR ALL STUDIES:*			
	Q1	Did the study address a clearly focussed issue?		
	Q2	Did the authors use an appropriate method to answer their question?		
	Q3	What are the results of this study? (Are they communicated clearly?)		
	Q4	How precise are the results?		
	Q5	Do you believe the results? (How believable are the results?)		
	Q6	Do the results of this study fit with other available evidence?		
	DEPENDING OF STUDY METHODOLOGY:(Q7: COMPLETE ONLY ONE OF THE TREE QUESTIONS)			
	Q7	Was the cohort recruited in an acceptable way?	***ONLY COMPLETE ONE OF THESE TREE QUESTIO[illegible]***	
	Q7	Were the cases/participants recruited in an acceptable way?		
	Q7	Was the follow up of subjects complete enough (if applicable)?		
	Q8	Was the study representative of the entire population from which subjects were recruited (if applicable)?		
	Q9	Can the results be applied to the local population (if applicable)?		
	Q10	Was the exposure and/or outcome accurately measured to minimise bias (if applicable)?		
	Q11	Have the authors identified and/or taken into account all important confounding factors in the design and/or analysis (if applicable)?		
	Q12	Was the follow up of subjects complete and/or long enough (if applicable)?		

QUALITY OF THE PAPER: APPRAISING SYSTEMATIC REVIEWS				0
Checklist for SYSTEMATIC REVIEWS (NO = 0; YES = 1)			TOTAL POINTS: REVIEWS	
complete this checlist when evaluating SYSTEMATIC REVIEWS	Q1	Did the study address a clearly focussed issue?		
	Q2	Did the authors use an appropriate method to answer their question?		
	Q3	Did the authors look for the right papers?		
	Q4	Were the important and relevant studies included?		
	Q5	Did the authors assess the quality of the included studies?		
	Q6	What are the overall results of the review? (Are they communicated clearly?)		
	Q7	How precise are the results?		
	DEPENDING ON THE REVIEW			
	Q8	If the results of the studies were combined or synthesised, was it reasonable to do this and/or was it done appropriately (if applicable)?		
	Q9	Can the results be applied to the local population (if applicable)?		
	Q10	Where all important outcomes considered (if applicable)?		
